# Elevated Estradiol and DHT Levels in the Prostatic Stroma as Key Drivers of Benign Prostatic Hyperplasia Pathogenesis

**DOI:** 10.3390/ijms27156785

**Published:** 2026-07-29

**Authors:** Tomislav Pejčić, Milka Jadranin, Siniša Đurašević, Milica Kalaba, Biljana Dojčinović, Milica Zeković, Uroš Bumbaširević, Darko Jovanović, Darko Laketić, Živoslav Tešić, Tomislav Tosti

**Affiliations:** 1 Faculty of Medicine, University of Belgrade, dr Subotića 8, 11000 Belgrade, Serbia; urosbu@gmail.com (U.B.); drdarkolaketic@gmail.com (D.L.); 2 Clinic of Urology, University Clinical Center of Serbia, Pasterova 2, 11000 Belgrade, Serbia; darkojov@gmail.com; 3 Department of Chemistry, University of Belgrade—Institute of Chemistry, Technology and Metallurgy, Njegoševa 12, 11158 Belgrade, Serbia; milka.jadranin@ihtm.bg.ac.rs (M.J.); biljana.matic@ihtm.bg.ac.rs (B.D.); tomislav.tosti@ihtm.bg.ac.rs (T.T.); 4 Faculty of Biology, University of Belgrade, Studentski trg 12–16, 11158 Belgrade, Serbia; sine@bio.bg.ac.rs; 5 Institute of General and Physical Chemistry, Studentski trg 12–16, 11158 Belgrade, Serbia; milicaffh@yahoo.com; 6 Centre of Research Excellence in Nutrition and Metabolism, Institute for Medical Research, National Institute of Republic of Serbia, University of Belgrade, Tadeuša Košćuška 1, 11000 Belgrade, Serbia; zekovicmilica@gmail.com; 7 Faculty of Chemistry, University of Belgrade, Studentski trg 12–16, 11158 Belgrade, Serbia; ztesic@chem.bg.ac.rs

**Keywords:** benign prostatic hyperplasia, prostate enlargement, transition zone, estradiol, dihydrotestosterone, testosterone, intraprostatic steroids

## Abstract

While dihydrotestosterone (DHT) has traditionally been considered the principal steroid in benign prostatic hyperplasia (BPH) pathogenesis, the role of estradiol (E2) and its relationship with local androgen accumulation remain unclear. This study investigated whether transition zone (TZ) tissue concentrations of E2, testosterone, and DHT are associated with prostate enlargement. TZ tissue cores were collected from 80 men undergoing prostate biopsy. Patients were stratified by total prostate volume (TPV): small (<30 mL) and enlarged (≥30 mL) prostate groups. Steroids were quantified using liquid chromatography-high resolution mass spectrometry. Tissue concentrations of E2, testosterone, and DHT were significantly higher in enlarged prostates (E2: 0.031 vs. 0.012 ng/g; testosterone: 0.804 vs. 0.517 ng/g; DHT: 7.903 vs. 3.526 ng/g; all *p* < 0.05). Across the cohort, TPV strongly correlated with DHT (R = 0.870, *p* < 0.001), E2 (R = 0.845, *p* < 0.001), and testosterone (R = 0.817, *p* < 0.001). These findings demonstrate a strong association between local steroid accumulation and prostate enlargement, supporting the hypothesis that coordinated androgenic and estrogenic activity shapes the hormonal microenvironment driving BPH progression. This pattern aligns with a model of local steroidal hyperfunction that may amplify stromal–epithelial signaling in the enlarged prostate.

## 1. Introduction

Benign prostatic hyperplasia (BPH) is a highly prevalent age-associated disorder characterized by progressive enlargement of the prostate within the transition zone (TZ) and by variable degrees of stromal and epithelial proliferation. The anatomical division into cranial and caudal prostates in non-human primates suggests that the TZ, PZ, and central zone (CZ) in the human prostate are evolutionarily distinct biological compartments. Such a perspective provides a biological framework for understanding why BPH and prostate cancer (PCa) arise in different anatomical zones.

Although prostate enlargement does not invariably translate into lower urinary tract symptoms, increasing total prostate volume (TPV) is clinically relevant because it is associated with a higher risk of BPH progression, acute urinary retention, and the need for surgical intervention [1,2,3]. The biological mechanisms underlying this progressive growth remain incompletely understood, but several converging processes have been implicated, including altered local steroid metabolism, reactivation of stromal–epithelial signaling, growth factor production, inflammation, and age-related remodeling of the prostatic microenvironment [1,2,3,4,5,6,7]. Previous tissue-biochemical studies have also suggested differences in hormonal and elemental composition, including zinc accumulation [8,9].

**Figure 1:**
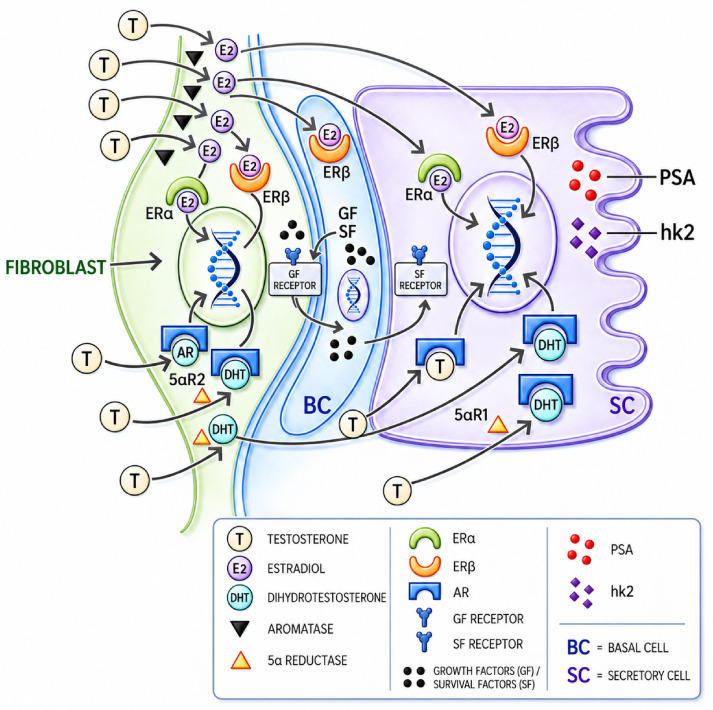
Stromal–epithelial interaction in benign prostatic enlargement. Schematic representation of reciprocal signaling between stromal and epithelial compartments in the prostate. Stromal cells contribute to local steroid metabolism and growth factor production, while epithelial cells respond through androgen receptor- and estrogen receptor-mediated pathways that regulate proliferation, differentiation, and secretory activity. Dysregulation of this stromal–epithelial network may contribute to transition-zone enlargement in BPH. Abbreviations: AR, androgen receptor; BC, basal cell; DHT, dihydrotestosterone; E2, estradiol; ERα, estrogen receptor alpha; ERβ, estrogen receptor beta; GF, growth factor; hK2, human kallikrein 2; PSA, prostate-specific antigen; SC, secretory cell; SF, stromal factor; T, testosterone; GF, growth factors; SF, survival factors. ChatGPT image tools (OpenAI, Chat GPT DALL·E, accessed July 2026.) were used solely for graphical refinement of an original author-created illustration. The authors reviewed and approved the final figure and take full responsibility for its scientific content.

The adult prostate is a hormonally responsive organ in which stromal and epithelial compartments interact continuously. Stromal fibroblasts and smooth muscle cells provide structural and paracrine support to the epithelium, while epithelial cells respond to stromally derived signals by modulating proliferation, differentiation, and secretory activity [1,2,4,5]. In BPH, the same developmental signaling framework may become reactivated or dysregulated, contributing to progressive tissue growth [1,2,3,4,5]. Growth factors such as fibroblast growth factors, vascular endothelial growth factor, insulin-like growth factors, transforming growth factor-β, and epidermal growth factor participate in this local network and can influence both stromal expansion and epithelial proliferation (Figure 1) [2,10,11,12,13,14].

Androgen signaling has traditionally occupied the central position in models of BPH pathogenesis. Testosterone enters prostate cells and is converted to dihydrotestosterone (DHT) by 5α-reductase isoenzymes, particularly within stromal cells [15,16,17,18,19]. DHT then activates the androgen receptor and supports androgen-dependent transcriptional programs involved in prostate growth and secretory function [2,20]. The clinical efficacy of 5α-reductase inhibitors further supports the importance of local DHT generation in men with enlarged prostates. However, the relationship between circulating testosterone and prostate growth is complex. Recent evidence indicates that intraprostatic steroid concentrations may not simply mirror circulating hormone levels [18,21,22,23,24,25,26,27]. These observations suggest that local steroid metabolism within the prostate, rather than systemic hormone concentrations alone, may be critical for understanding benign prostatic enlargement.

In addition to androgens, estrogens are increasingly recognized as biologically important regulators of the male reproductive tract. Estradiol (E2), the most potent endogenous estrogen, contributes to several physiological processes in males, including reproductive function, libido, and spermatogenesis [28,29,30]. Estrogens are generated from androgen precursors, including testosterone and androstenedione, through aromatization mediated by cytochrome P450 aromatase, encoded by CYP19A1 [31]. In the normal human prostate, aromatase expression has been described predominantly in stromal cells, whereas altered localization and expression patterns have been reported in malignant prostate tissue [32,33,34]. Locally produced estrogens can therefore act in a paracrine or intracrine manner within the prostate and may influence both normal tissue homeostasis and pathological growth [35,36,37].

The biological effects of estrogens in the prostate are mediated primarily through estrogen receptor alpha (ERα) and estrogen receptor beta (ERβ), which have distinct tissue distributions and functional consequences [38,39,40,41,42,43,44]. ERα is predominantly associated with stromal signaling and has generally been linked to proliferative and pro-inflammatory responses, whereas ERβ is expressed in both stromal and epithelial compartments and is often considered antiproliferative, differentiating, and potentially protective [42,43,44,45,46,47,48,49]. The balance between ERα- and ERβ-mediated signaling may therefore be particularly important in determining whether estrogen exposure promotes or restrains prostatic growth. Estrogen signaling is not limited to classical nuclear receptor pathways; it may also involve membrane-associated receptors, ligand-independent receptor activation, and interaction with other transcriptional and kinase signaling cascades [50,51,52,53,54,55].

**Figure 2:**
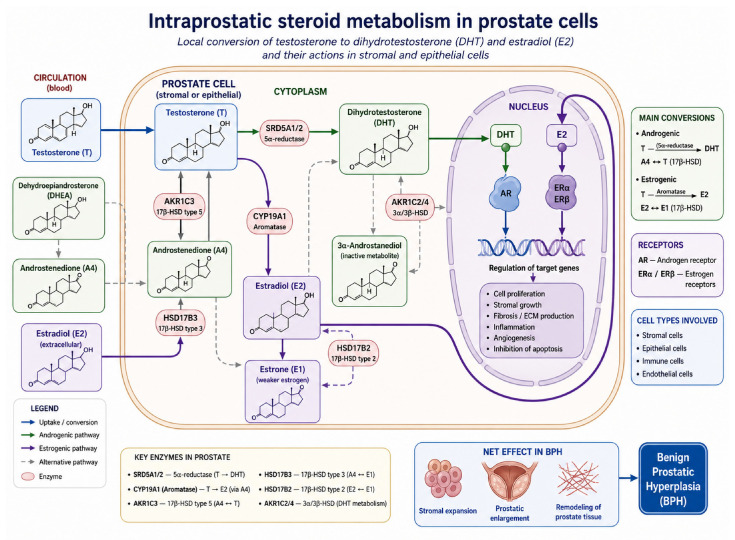
Intraprostatic steroid metabolism in prostate cells. Schematic overview of local steroid-conversion pathways linking androgenic and estrogenic signaling in prostate tissue. Testosterone may be converted to DHT by 5α-reductase or to E2 through aromatase activity, while additional intracrine pathways involve DHEA, androstenedione, estrone, and 3α-androstanediol. Coordinated accumulation of testosterone, DHT, and E2 may contribute to a local hormonal microenvironment associated with prostate enlargement. Abbreviations: A4, androstenedione; AR, androgen receptor; CYP19A1, aromatase; DHEA, dehydroepiandrosterone; DHT, dihydrotestosterone; E1, estrone; E2, estradiol; ERα, estrogen receptor alpha; ERβ, estrogen receptor beta; HSD, hydroxysteroid dehydrogenase; SRD5A1/2, steroid 5α-reductase type 1/2; T, testosterone. ChatGPT image generation tools (OpenAI, DALL·E, accessed July 2026.) were used to assist in the graphical creation of the schematic based on author provided scientific information, conceptual guidance, and detailed textual instructions. The authors defined the biological content of the schematic, including the pathways, molecular interactions, labels, and layout. The resulting image was reviewed and approved by the authors, who take full responsibility for the accuracy and integrity of the figure.

Several experimental and clinical observations support a role for estrogenic signaling in BPH (Figure 2). Enhanced stromal ERα activity can promote stromal proliferation and inflammatory gene expression, while changes in ERβ expression have been associated with altered epithelial differentiation and loss of growth restraint [12,47,56,57,58,59,60,61,62]. In BPH tissue, increased stromal activity may also be accompanied by activation of receptor tyrosine kinase pathways, chemokine signaling, and inflammatory mediator release, all of which can reinforce fibroblast proliferation and epithelial growth [6,7]. In this context, the combined action of androgens and estrogens may be more relevant than the isolated effect of either steroid class. Testosterone can serve as a local precursor for both DHT and E2, thereby linking androgenic and estrogenic signaling within the same tissue microenvironment [15,16,17,18,19,31,63,64,65,66,67]. Collectively, these observations suggest that the unresolved question is no longer whether sex steroids participate in BPH biology, but rather how their coordinated intraprostatic distribution relates to clinically relevant prostate enlargement.

Despite this biological rationale, the clinical significance of intraprostatic estrogens in BPH remains insufficiently defined. Earlier studies demonstrated accumulation of estrogens and androgens within the stromal compartment of hyperplastic prostates, but many of these investigations relied on radioimmunoassay-based methods and surgical tissue specimens, which may be affected by tissue handling, ischemia, thermal injury, zonal heterogeneity, and differences in analytical sensitivity [68,69,70,71]. More recent studies using mass spectrometry-based approaches have improved steroid quantification in prostate tissue, but important methodological variability remains, particularly with respect to tissue source, anatomical zone, histological context, and whether benign, hyperplastic, and malignant tissue compartments are analyzed separately [25,68,69,70,71,72,73,74,75].

A more precise assessment of local steroid concentrations in biopsy-derived transition-zone tissue may help clarify whether prostate enlargement is associated with coordinated accumulation of E2, testosterone, and DHT. This approach is particularly relevant because BPH arises predominantly in the TZ, while many previous studies have used surgical specimens, mixed prostatic zones, or non-standardized tissue sampling strategies [1,2,3,68,69,70,71,73,74,75]. Moreover, evaluating E2 together with testosterone and DHT allows local estrogenic activity to be interpreted within the broader steroidogenic context of the prostate rather than as an isolated hormonal variable.

The present study investigated tissue concentrations of E2, testosterone, and DHT in TZ prostate biopsy samples obtained during transrectal ultrasound-guided biopsy. Patients were stratified according to TPV using a clinically relevant 30 mL threshold, and tissue hormone concentrations were compared between men with smaller and enlarged prostates. We further evaluated the relationships between TPV and each measured steroid, as well as the degree of coordinated accumulation among E2, testosterone, and DHT. We hypothesized that enlarged prostates would exhibit higher TZ tissue concentrations of E2, testosterone, and DHT, consistent with coordinated intraprostatic steroid accumulation and a shift toward a local hormonal milieu conducive to stromal–epithelial activation and progressive benign prostatic enlargement.

## 2. Results

### 2.1. Transition-Zone Tissue Steroid Concentrations According to Prostate Volume

Concentrations of all three measured steroids were significantly higher in transition-zone biopsy tissue from men with enlarged prostates. The mean E2 concentration was more than two-fold higher in the TPV ≥ 30 mL group than in the TPV < 30 mL group (0.034 ± 0.018 vs. 0.014 ± 0.007 ng/g, *p* < 0.0001). Similarly, mean testosterone concentration was significantly higher in enlarged prostates (0.906 ± 0.312 vs. 0.533 ± 0.154 ng/g, *p* < 0.0001), as was mean DHT concentration (8.671 ± 3.525 vs. 3.663 ± 1.125 ng/g, *p* < 0.0001).

**Figure 3:**
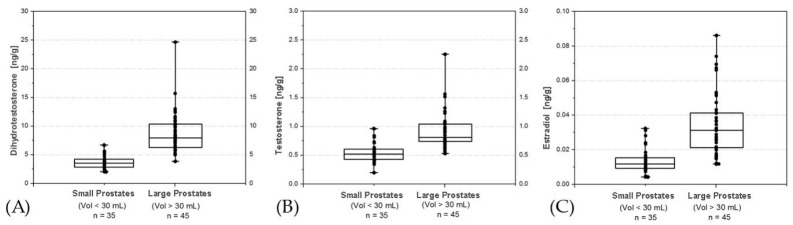
Differences in tissue concentrations of DHT (**A**), testosterone (**B**), and estradiol (**C**) between patients with small and enlarged prostates. (**A**) DHT: in individuals with small prostates, DHT values are clustered and low, whereas the group with larger prostates demonstrates a higher median and a wider range of values (*p* < 0.001). (**B**,**C**) Testosterone, estradiol: a similar trend of statistically significant differences is observed (*p* < 0.001), albeit with a slightly greater overlap of individual data points between the quartiles of both groups compared to DHT. The horizontal black line within the box represents the median, the box boundaries represent the interquartile range (IQR), while the individual grey dots illustrate the actual, exact values of each patient in the cohort. The *p*-values indicate the statistical significance of the differences determined by the Mann–Whitney U test (*p* < 0.001 for all three hormones).

The Mann–Whitney U test confirmed significant differences between the small and enlarged prostate groups for E2 (U = 155.50, *p* < 0.001), testosterone (U = 135.50, *p* < 0.001), and DHT (U = 41.00, *p* < 0.001). Descriptive data for tissue hormone concentrations according to TPV category are shown in Table 1, and their distribution is illustrated in Figure 3.

### 2.2. Relationship Between Total Prostate Volume and Transition-Zone Tissue Steroid Concentrations

Correlation and regression analyses demonstrated significant positive associations between TPV and tissue steroid concentrations. Across the entire cohort, TPV showed a strong positive correlation with DHT (R = 0.870, *p* < 0.001), followed by E2 (R = 0.845, *p* < 0.001) and testosterone (R = 0.817, *p* < 0.001).

When the analysis was performed separately according to TPV category, different patterns were observed. In the TPV < 30 mL group, E2 and testosterone concentrations were not significantly associated with prostate volume. For E2, the correlation coefficient was R = 0.259, with a regression slope of 0.0004 and *p* = 0.134. For testosterone, the correlation coefficient was R = 0.244, with a regression slope of 0.007 and *p* = 0.160. In contrast, DHT showed a significant positive association with TPV even in the small prostate group (R = 0.612, slope = 0.155, *p* < 0.001).

In the TPV ≥ 30 mL group, all three steroids showed significant positive associations with prostate volume. E2 demonstrated the strongest correlation with TPV (R = 0.818, slope = 0.0003, *p* < 0.001), followed by testosterone (R = 0.641, slope = 0.007, *p* < 0.001) and DHT (R = 0.638, slope = 0.082, *p* < 0.001).

### 2.3. Coordinated Accumulation of Intraprostatic Steroids

**Figure 4:**
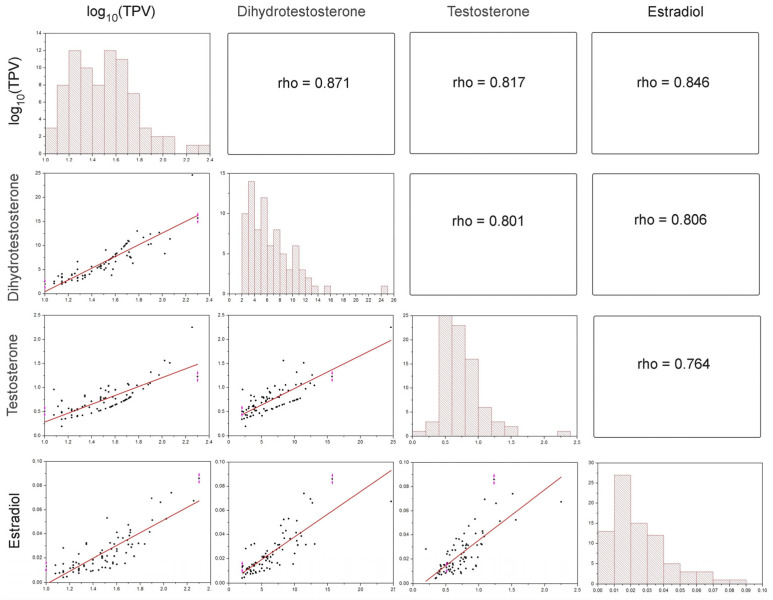
Correlation matrix of hormonal parameters and total prostate volume (TPV). The main diagonal displays frequency distribution histograms for each variable: total prostate volume on a logarithmic scale (log10(TPV)), dihydrotestosterone (DHT), testosterone, and estradiol (E2). The lower-left triangle presents scatterplots with regression lines illustrating the linear relationship between variable pairs. The upper-right triangle shows the Spearman rank correlation coefficients (ρ) along with their corresponding statistical significance levels (*p*-value).

Correlation-matrix analysis further supported a coordinated pattern of steroid accumulation in TZ tissue. TPV was positively associated with each measured hormone, and strong positive relationships were also observed among E2, testosterone, and DHT. These findings indicate that prostate enlargement is accompanied not by an isolated increase in a single steroid, but by a broader increase in local androgenic and estrogenic steroid concentrations. The correlation structure among TPV, E2, testosterone, and DHT is shown in Figure 4.

### 2.4. Effect Sizes and Sensitivity Analysis of Between-Group Hormonal Differences

Given the non-normal distribution of the data, between-group effect sizes were primarily quantified using Cliff’s Δ with bias-corrected and accelerated (BCa) bootstrap 95% confidence intervals based on 100,000 resamples and a fixed random seed (12345), while Cohen’s d with 95% confidence intervals was additionally calculated as a supportive standardized measure of the difference between group means (Table 2).

Large between-group effects were observed for all three hormones. The strongest distributional separation was found for DHT, with Cliff’s Δ = 0.948 and a BCa bootstrap 95% CI of 0.845–0.985. Large effects were also observed for testosterone (Δ = 0.828, BCa bootstrap 95% CI 0.650–0.925) and E2 (Δ = 0.803, BCa bootstrap 95% CI 0.625–0.906). All confidence intervals were entirely above zero, indicating a consistent predominance of higher hormone concentrations in the TPV ≥ 30 mL group relative to the TPV < 30 mL group of patients. Moreover, even the lower confidence limits remained within the range conventionally interpreted as a large effect.

The complementary standardized mean differences were also large and directionally consistent with the Cliff’s delta estimates. Cohen’s d was 1.821 for DHT (95% CI 1.291–2.343), 1.459 for testosterone (95% CI 0.958–1.953), and 1.407 for E2 (95% CI 0.910–1.897). Because the hormone distributions were asymmetric and included extreme values, particularly for DHT and E2, these Cohen’s d estimates were interpreted as supportive rather than primary measures of effect magnitude.

A sensitivity analysis further indicated that, with 35 patients in the TPV < 30 mL group and 45 patients in the TPV ≥ 30 mL group, a two-sided α level of 0.05, and an independent-samples design, the available sample provided 80% power to detect a standardized between-group effect of approximately Cohen’s d = 0.639 or greater and 90% power to detect an effect of approximately d = 0.740 or greater. Thus, the study was adequately powered to detect moderate-to-large standardized between-group differences.

Overall, the concordance between the large Cliff’s delta estimates and the complementary Cohen’s d values demonstrates substantial separation between the two patient groups for DHT, testosterone, and E2, with the primary interpretation based on the distribution-free Cliff’s delta estimates and their bootstrap confidence intervals.

## 3. Discussion

The present study demonstrates that TZ prostate biopsy tissue from men with enlarged prostates contains significantly higher concentrations of E2, testosterone, and DHT than tissue from men with smaller prostates. This pattern was consistent across all three measured steroids and was accompanied by strong positive correlations between TPV and local tissue hormone concentrations. Among the steroids investigated, DHT showed the strongest overall correlation with TPV, followed by E2 and testosterone. Consistent with our previous work based on a different patient cohort, TZ tissue hormone concentrations did not differ significantly based on the presence of prostate cancer detected in the PZ [9].

These findings support the concept that benign prostatic enlargement is associated not only with local DHT accumulation, but also with a coordinated increase in intraprostatic estrogenic and androgenic steroid concentrations. Testosterone may provide a substrate for simultaneous androgen receptor- and estrogen receptor-mediated signaling. Although the cross-sectional design of this study does not allow causal inference, the observed hormonal profile is consistent with a model in which local steroidal hyperfunction may contribute to the microenvironment that supports stromal–epithelial activation and progressive prostate enlargement.

An important aspect of these findings is that steroid quantification was performed in TZ biopsy tissue, the anatomical region most directly related to BPH, rather than in tumor-directed diagnostic cores. Therefore, the observed hormonal pattern should be interpreted primarily in relation to prostate volume and transition-zone enlargement, not as a reflection of malignant tissue status. At the same time, because the biopsy cores were not histologically micro dissected, the measured steroid concentrations represent tissue-level transition-zone values rather than compartment-specific stromal or epithelial measurements.

### 3.1. Relationship with Previous Studies on Intraprostatic Estrogens

The role of DHT in BPH has been extensively investigated, whereas the clinical and biological significance of intraprostatic estrogens has received comparatively less attention. Early studies suggested that estrogens accumulate within hyperplastic prostate tissue and may be particularly relevant to the stromal compartment. Kozak et al. [68] reported that E2 concentrations were higher in BPH tissue than in plasma and that the nuclei of stromal cells represented a site of pronounced estrogen accumulation. They concluded that E2 may contribute to stromal growth in BPH [68], and subsequent studies demonstrated nuclear accumulation of both E2 and DHT within stromal cells [69], Stone et al. [70] also reported increased estrogen formation in periurethral tissue from patients with BPH compared with tissue from patients without BPH (Table 3).

In a later study, Krieg et al. [71] compared normal and hyperplastic prostate tissue and found that E2 levels increased in the stroma of BPH tissue, whereas epithelial E2 concentrations were relatively lower (Table 3). These findings suggested an age-related shift in the local estrogen-androgen balance, particularly within the stromal compartment of the hyperplastic prostate [71]. Together, these early investigations provided a biologically plausible basis for considering E2 as an active participant in BPH rather than merely a systemic background hormone. However, interpretation of these studies is limited by the analytical methods available at that time, differences in tissue processing, and the use of surgical specimens.

More recent studies have used mass spectrometry-based approaches to improve the sensitivity and specificity of steroid measurements in prostate tissue. Arai et al. [73] developed a liquid chromatography–mass spectrometry method for measuring E2 in prostatic tissue and reported that intraprostatic E2 concentrations increased with age. Cook et al. [25] analyzed circulating and intraprostatic sex steroid concentrations in a large multicenter cohort and found no consistent correlation between serum and tissue steroid levels, emphasizing that local prostate hormone concentrations cannot be reliably inferred from circulating measurements. This observation is particularly relevant for BPH pathophysiology, because it supports the idea that the prostate may develop a distinct local steroidal microenvironment.

The STERPROSER and STERKPROSER studies further highlighted the complexity of intraprostatic steroid distribution (Table 3). Neuzillet et al. [74] analyzed non-cancerous prostate tissue and reported differences in tissue steroid concentrations according to prostate weight, although tissue sampling was performed on surgical tissue without consistently defining the specific prostatic zone. Meunier et al. [75] later showed that steroid concentrations differed between cancerous and benign prostate tissues and between central and peripheral prostate regions, underscoring the importance of anatomical zone, tissue context, and sampling strategy when interpreting intraprostatic hormone levels.

Our findings are broadly consistent with the earlier concept that estrogens and androgens accumulate within enlarged or hyperplastic prostate tissue, but they also extend this concept in two important ways. First, E2, testosterone, and DHT were measured together in transition-zone biopsy tissue, allowing estrogenic and androgenic activity to be evaluated within the same local steroidal framework. Second, tissue acquisition by transrectal ultrasound-guided biopsy minimized several potential artifacts associated with surgical or transurethral resection specimens, including ischemic delay, thermal injury, and uncertainty regarding immediate tissue handling. These methodological features may be particularly important when studying low-abundance steroids such as E2.

### 3.2. Testosterone Accumulation as a Prerequisite for Local Steroidal Hyperfunction in Prostate Enlargement

The coordinated increase in E2, testosterone, and DHT observed in enlarged prostates suggests that benign prostatic growth is accompanied by a broader alteration in local steroid metabolism rather than by isolated DHT accumulation alone. In this context, testosterone occupies a central upstream position because it can serve as a precursor for both DHT, through 5α-reductase activity, and E2, through aromatase-mediated conversion [15,16,17,18,19,31]. The concurrent elevation of all three steroids in transition-zone tissue therefore supports a model in which local testosterone accumulation provides the substrate for parallel amplification of androgenic and estrogenic signaling within the prostate microenvironment.

We propose that sustained intraprostatic testosterone accumulation may creates the biochemical conditions for increased generation of both DHT and E2. DHT may then enhance androgen receptor-mediated transcriptional activity, whereas E2 may stimulate ERα-associated stromal pathways that promote fibroblast proliferation, inflammatory activation, extracellular matrix remodeling, growth factor release, and stromal–epithelial paracrine signaling [19,43,44,45,46,47,48,49].

The mechanism responsible for increased intraprostatic testosterone availability in BPH remains unresolved. Several non-mutually exclusive explanations may be considered. One possibility is that testosterone is insufficiently eliminated or metabolically cleared within prostate cells. Reduced elimination of testosterone in the form of testosterone glucuronide has been described in malignant prostate tissue, although this mechanism has not yet been clearly demonstrated in BPH [76,77]. Another possibility is that the enzymatic machinery required for testosterone conversion is not limited, allowing accumulated testosterone to be continuously available for conversion into DHT and E2 [78]. In addition, de novo testosterone synthesis has been demonstrated in human prostate cell lines and prostate biopsies, and although this pathway has been more extensively considered in the context of prostate cancer, it may also be biologically relevant to benign prostate enlargement [79].

A further mechanism may involve the intracellular sequestration of testosterone. This cellular accumulation is likely mediated by enhanced interaction with the androgen receptor (AR) or receptor fragments, consistent with the elevated AR expression observed in BPH tissue [80]. In this scenario, testosterone accumulation would not only increase the substrate pool for DHT and E2 generation but would also intensify AR-dependent signaling within stromal and epithelial compartments.

Sex hormone-binding globulin (SHBG) may also contribute to local steroid handling in the prostate. Although classically regarded as a circulating transport protein produced by the liver, SHBG is also locally synthesized in human prostate tissue and may act as an autocrine or paracrine regulator [81]. SHBG-dependent signaling triggers rapid intracellular responses in prostate cells. In cancer models, this pathway alters testosterone action and metabolism, thereby inhibiting the elimination of testosterone from the cell [82,83]. Whether analogous SHBG-related mechanisms participate in testosterone accumulation in BPH remains to be established, but they provide a plausible additional layer of local hormonal regulation.

This model is also compatible with the slow natural history of BPH. Microscopic BPH changes may begin decades before clinically relevant prostate enlargement becomes evident, and age-related benign prostate growth has long been recognized as a progressive process [3,84]. It is therefore plausible that gradual testosterone accumulation begins before overt macroscopic enlargement and proceeds in parallel with stromal–epithelial reactivation, growth factor signaling, inflammation, and progressive tissue remodeling. Over time, increased local testosterone availability may drive greater production of both DHT and E2, thereby establishing a self-reinforcing hormonal microenvironment that promotes stromal proliferation, epithelial proliferation, and enlargement of the TZ.

The present findings support this hypothesis by showing that TZ tissue from enlarged prostates contains significantly higher concentrations of testosterone, DHT, and E2 than tissue from smaller prostates, and that all three steroids are positively associated with TPV. The observation that E2 showed strong correlation with TPV does not necessarily indicate that E2 is the initiating cause of prostate enlargement. Rather, it suggests that estrogenic signaling may become increasingly important once local testosterone accumulation has generated sufficient substrate for aromatase-mediated E2 production. In smaller prostates, DHT was already significantly associated with TPV, whereas E2 and testosterone were not. In enlarged prostates, however, all three steroids showed significant positive associations with TPV. This pattern is consistent with a model in which DHT-related androgenic signaling may be involved early, while progressive enlargement is characterized by coordinated testosterone, DHT, and E2 accumulation and the emergence of local prostatic hormonal hyperfunction.

Taken together, these data suggest that BPH-associated prostate enlargement should not be conceptualized exclusively as a consequence of isolated DHT excess. Instead, enlarged transition-zone tissue appears to be characterized by coordinated steroidal accumulation, in which testosterone may act as the upstream substrate sustaining both DHT-dependent androgenic signaling and E2-dependent estrogenic signaling. This integrated hormonal model provides a biologically coherent explanation for the observed relationship between tissue steroid concentrations and prostate volume and supports further investigation of testosterone retention, local steroidogenic enzyme activity, AR signaling, SHBG-mediated regulation, and estrogen receptor balance in the pathogenesis of benign prostatic hyperplasia.

### 3.3. Methodological and Translational Implications

A major methodological strength of this study is the use of transition-zone biopsy tissue for steroid quantification, i.e., tissue where BPH exclusively arises. Many previous studies of intraprostatic steroids used tissue obtained during radical prostatectomy, cystoprostatectomy, open prostatectomy, or transurethral resection. Such specimens may contain mixed anatomical zones or tissue regions selected after surgical manipulation, and they may be affected by variable ischemic intervals, electrocautery, coagulation injury, thermal artifacts, or delayed freezing. In contrast, biopsy-derived tissue can be rapidly transferred and frozen, thereby reducing pre-analytical variability. This feature is particularly important when measuring low-abundance steroids such as E2, where tissue handling and analytical sensitivity may substantially influence the interpretation of local hormone concentrations.

At the same time, this sampling strategy requires careful interpretation. The transition-zone biopsy cores analyzed in this study were not subjected to histological microdissection; therefore, the measured steroid concentrations should be understood as tissue-level concentrations in TZ biopsy material rather than as measurements from a purified stromal or epithelial compartment. This distinction is important because the TZ contains variable proportions of stromal cells, glandular epithelium, vascular structures, inflammatory cells, and extracellular matrix. Nevertheless, because BPH is fundamentally a transition-zone process involving both stromal and epithelial components, biopsy-derived TZ tissue provides a clinically relevant substrate for evaluating local hormonal changes associated with prostate enlargement.

The lack of significant differences in transition-zone tissue steroid concentrations according to diagnostic prostate cancer status further supports the interpretation that the main hormonal signal in this study was related to prostate volume. This point is methodologically important because the cohort included men undergoing biopsy for suspected prostate cancer; however, steroid quantification was not performed in tumor-directed diagnostic cores, and the primary comparison was not based on malignant versus benign tissue. Rather, steroid concentrations were measured in additional transition-zone biopsy cores and evaluated across prostate-volume categories. Therefore, prostate cancer status should be interpreted as a patient-level clinical and histopathological variable, not as evidence of tissue-compartment overlap in the steroid measurements. Nevertheless, because some patients had markedly elevated PSA values and clinically advanced disease, residual systemic or patient-level effects cannot be entirely excluded and should be considered when interpreting the data. The cross-sectional nature of the investigation precludes conclusions regarding temporal relationships or causality, and it remains uncertain whether coordinated intraprostatic steroid accumulation precedes prostate enlargement or develops as a consequence of progressive tissue remodeling and altered local steroidogenic capacity. An alternative interpretation that merits consideration is that the observed hormonal profile may reflect the biological consequences of increasing prostate volume itself rather than representing the initiating event in BPH pathogenesis. Distinguishing between these possibilities will require longitudinal and mechanistic investigations. In addition, because the study population consisted of men undergoing prostate biopsy for suspected prostate cancer, the generalizability of the findings to the broader population of men with uncomplicated BPH warrants further investigation.

The present findings may also have implications for how the hormonal biology of BPH is conceptualized. Current pharmacological treatment directed at prostate volume reduction relies primarily on inhibition of 5α-reductase, thereby reducing local DHT synthesis. However, if enlarged prostates are characterized by simultaneous increases in testosterone, DHT, and E2, then BPH-associated hormonal activity may not be fully explained by DHT alone. A broader model that incorporates testosterone accumulation, DHT-dependent androgenic signaling, local E2 generation, stromal ERα activation, ERβ-mediated counter-regulation, and stromal–epithelial paracrine amplification may better reflect the complexity of benign prostatic growth.

These results do not provide direct evidence that estrogen-modulating therapies should be used in men with BPH. The study did not evaluate clinical outcomes, receptor expression, aromatase activity, response to 5α-reductase inhibitors, or longitudinal progression. Nevertheless, the association between E2 accumulation and prostate enlargement supports further investigation of estrogen-related pathways as contributors to BPH biology. Future studies should assess whether transition-zone tissue E2 concentrations correlate with ERα/ERβ expression, aromatase and 5α-reductase activity, androgen receptor signaling, stromal-to-epithelial ratio, inflammatory infiltrates, fibrosis, lower urinary tract symptoms, prostate growth rate, and response to medical therapy.

The biopsy-based approach used in this study may therefore be relevant for future translational research. Measurement of local steroid concentrations in transition-zone biopsy tissue could, in principle, help define hormonal phenotypes of prostate enlargement. Such phenotyping may be particularly useful if combined with histology, immunohistochemistry, gene-expression analysis, serum hormone profiling, urinary or prostatic secretion biomarkers, and longitudinal clinical follow-up. In this context, urinary PSA has previously been investigated as a potential marker of prostatic secretory activity and BPH progression, suggesting that local steroid profiling could eventually be integrated with complementary urinary biomarkers in future translational studies [85,86]. However, this concept remains investigational and should not be interpreted as an immediately applicable diagnostic or therapeutic strategy. Rather, it provides a framework for future studies aimed at determining whether coordinated intraprostatic accumulation of testosterone, DHT, and E2 identifies biologically distinct forms of prostate enlargement and whether these hormonal phenotypes have predictive value for BPH progression or treatment response.

## 4. Materials and Methods

### 4.1. Study Design and Patient Cohort

This observational study included 80 men who underwent TRUS-guided prostate biopsy at the Clinic of Urology, University Clinical Centre of Serbia, during 2017 because of clinical suspicion of PCa, based on an elevated serum PSA concentration and/or an abnormal digital rectal examination. In a subset of patients with PSA values within the metastatic range, biopsy was performed to obtain histopathological confirmation before the initiation of androgen deprivation therapy. At the time of patient recruitment, multiparametric magnetic resonance imaging of the prostate was not routinely implemented in the diagnostic pathway at our institution.

The study was designed as a biopsy-based analysis of TZ prostate tissue rather than as a comparison between malignant and benign tumor compartments. Inclusion was limited to patients for whom additional TZ biopsy material was available and suitable for steroid quantification. Therefore, the study cohort represents a selected biopsy-based subgroup of patients undergoing prostate biopsy at our institution, rather than the entire population evaluated during the study period. All eligible samples fulfilling these predefined criteria were included in the analysis. Standard diagnostic biopsy cores, obtained predominantly from the PZ, were used for routine histopathological examination. In contrast, the additional cores used for steroid quantification were collected from the TZ, the anatomical compartment directly involved in BPH. Because the TZ cores were not separately subjected to histological examination, hormone concentrations are reported as TZ biopsy tissue concentrations rather than as compartment-specific stromal or epithelial values.

Before biopsy, medication history was reviewed, with particular attention to drugs that could influence PSA values or intraprostatic steroid metabolism, including 5α-reductase inhibitors, androgen deprivation therapy, antiandrogens, exogenous androgens, and other hormonal treatments. Relevant clinical variables, including age, serum PSA concentration, digital rectal examination findings, total prostate volume, were recorded for analysis. The demographic and clinical characteristics of the included patients are summarized in Supplementary Table S1.

TPV was determined by TRUS at the time of biopsy. Patients were stratified according to a clinically relevant TPV threshold of 30 mL. Men with TPV < 30 mL were assigned to the small prostate group, whereas men with TPV ≥ 30 mL were assigned to the enlarged prostate group. This threshold was selected because a prostate volume of approximately 30 mL is commonly used to identify clinically meaningful prostate enlargement and an increased risk of BPH progression, and it is also relevant for therapeutic decision-making in men considered for 5α-reductase inhibitor therapy.

### 4.2. Prostate Biopsy and Transition-Zone Tissue Sampling

All prostate biopsies were performed under TRUS guidance by experienced urologists. Standard diagnostic biopsy consisted of 12 to 18 cores obtained predominantly from the PZ and submitted for routine histopathological examination. After written informed consent had been obtained, two additional biopsy cores were collected from the TZ specifically for steroid analysis. These cores were anatomically and analytically distinct from the standard diagnostic biopsy cores used for histopathological assessment of prostate cancer status. During TZ sampling, care was taken to avoid areas suspected of tumor infiltration.

Immediately after collection, TZ tissue cores intended for chemical analysis were placed into pre-weighed 1.5 mL Eppendorf tubes, rapidly frozen in liquid nitrogen, and stored at −70 °C until analysis. The rapid transfer and freezing of biopsy material were intended to minimize post-sampling degradation and to avoid the ischemic and thermal artifacts.

### 4.3. Ethical Approval and Informed Consent

The study was conducted in accordance with institutional and ethical requirements. Written informed consent was obtained from all participants before the collection of additional transition-zone tissue cores for steroid analysis. The study received institutional approval from the Clinic of Urology, University Clinical Centre of Serbia in Belgrade (Protocol No. 1033, 24 December 2025) and approval from the Ethics Committee of the University Clinical Centre of Serbia in Belgrade (Decision No. 1880/84, 25 December 2025).

### 4.4. Chemical Analysis

#### 4.4.1. Reagents

DHT (D-073; 1 mg/mL in methanol, ampule of 1 mL, certified reference material) and 16,16,17-trideuterated dihydrotestosterone-D3 (D-077 100 μg/mL in methanol, ampule of 1 mL, certified reference material, Cerilliant isotopic purity 98 atom%), 17β-estradiol (3301-1GM ≥ 97% (HPLC), solid, estrogenic hormone, Calbiochem), 17β-estradiol-d3 (E2-d3; 491187, 98 atom % D, 99% (CP)), testosterone (86500-1G purity ≥ 99.0% (HPLC), androgenic steroid, powder), and 16,16,17-trideuterated testosterone-D3 (T-046 100 μg/mL in acetonitrile, ampule of 1 mL, certified reference material, Cerilliant), used as internal standards, were purchased from Sigma-Aldrich (Steinheim, Germany). The derivatization reagent dansyl chloride (03641 LiChropur™, ≥99.0% (HPLC)) was also obtained from Sigma-Aldrich (Steinheim, Germany). Acetonitrile (1.00029 ≥99.9% (GC), suitable for LC/MS, LiChrosolv), formic acid (33015 puriss. p.a., ACS reagent, reag. Ph. Eur., ≥98%), ethyl acetate (34858 suitable for HPLC, ≥99.7%), and methanol (1.06018 ≥99.8% (GC), HPLC grade, suitable for HPLC, LiChrosolv) were from Merck (Darmstadt, Germany). Ultrapure water obtained by a Thermofisher TKA MicroPure water purification system Niederelbert, Germany, purification system was used for preparation of aqueous solutions. Strata-X solid-phase extraction (SPE) cartridges (3 mL, 60 mg) were obtained from Phenomenex (Torrance, CA, USA). The ESI-L Low Mix Tuning Solution (G1969-85000), API Reference Mass Solution (G1969-85001), and ZORBAX Eclipse Plus C18 analytical column (100 × 2.1 mm i.d., 1.8 μm particle size) were purchased from Agilent Technologies (Waldbronn, Germany).

#### 4.4.2. Standard Solutions

Standard stock solutions of 17β-estradiol (E2), DHT, and testosterone, were prepared in methanol at a concentration of 1000 ng/mL. Working solutions were obtained by serial dilution of the stock solutions with methanol to concentrations corresponding to the expected steroid levels in the analyzed samples. Calibration standards were prepared by further dilution of the working solutions with methanol. The calibration ranges were 1–25 ng/mL for DHT, 0.01–0.5 ng/mL for E2, and 0.1–5 ng/mL for testosterone. Quality control (QC) solutions used to monitor analytical performance were prepared by diluting the standards to concentrations ranging from 0.9 to 100 ng/mL, depending on the expected analyte levels in the samples. Identification and quantification of the steroids were carried out using authentic analytical standards.

Analyte identification was confirmed by direct comparison of retention times and mass spectra with those of the corresponding reference standards. Under the optimized chromatographic conditions, testosterone, DHT, and E2 eluted at 8.42, 9.23, and 10.49 min, respectively. Quantification was based on the protonated molecular ions at *m*/*z* 289.2162 for testosterone, *m*/*z* 291.2319 for DHT, and *m*/*z* 506.2360 for dansyl-derivatized E2. The derivatization procedure applied for E2 analysis did not interfere with the determination of testosterone or DHT, enabling their simultaneous analysis without analytical cross-interference.

#### 4.4.3. Validation of the Method

Method validation parameters, including limits of detection (LOD), limits of quantification (LOQ), and recovery at three concentration levels, were determined accordance with the methodology reported by Pejčić at al. [9]. The validation parameters described in detail in Supplementary Materials, are summarized in Table S2.

#### 4.4.4. Accuracy and the Precision of the Method

The accuracy and precision of the method were performed as described in our previously published study [9], and the results are given in Table S3, while the detail methodology is provided in the Supplementary Materials.

#### 4.4.5. Biological Samples

The tissue samples were subsequently weighed, finely minced using surgical scissors, homogenized in glass tissue grinder in ice cold bath for 10 min, and extracted with 500 μL of ethyl acetate in an ice-cooled ultrasonic bath for 15 min. The resulting mixture was centrifuged at 17,530× *g* for 15 min at 4 °C. The supernatant was carefully transferred to a clean test tube and evaporated to dryness under a gentle stream of nitrogen at ambient temperature (20 ± 2 °C). The remaining residue was re-extracted with 500 μL of methanol and processed under identical conditions.

#### 4.4.6. Solid Phase Extraction

The obtained residue was reconstituted in 1000 μL of 10% methanol and loaded onto a Strata-X SPE cartridge, which had been conditioned with 3 mL of methanol followed by 9 mL of ultrapure water. After sample loading, the cartridge was washed with 3 mL of 5% methanol to remove interfering matrix components. The target analytes were then eluted using 90% methanol.

The eluate was evaporated to dryness under a gentle stream of nitrogen. The final residue was re-dissolved in 500 μL of methanol containing 0.1% formic acid and transferred to LC vials for LC–HRMS analysis. The prepared extract was divided into two aliquots: one was used for testosterone, and DHT analysis, while the other was reserved for E2 determination.

#### 4.4.7. Liquid Chromatography-High Resolution Mass Spectrometry (LC–HRMS)

Chromatographic separation was carried out using an Agilent 1200 high-performance liquid chromatography (HPLC) system (Agilent Technologies, Waldbronn, Germany) equipped with a binary pump, online vacuum degasser, autosampler, and diode array detector (DAD). Analyte separation was achieved on a Zorbax Eclipse Plus C18 analytical column (100 × 2.1 mm i.d., 1.8 μm particle size), maintained at 40 °C. Data acquisition and processing were performed using MassHunter Workstation software (version A.02.02, Agilent Technologies). The HPLC system was coupled to an Agilent 6120 Time-of-Flight LC–HRMS system operating with electrospray ionization (ESI) in positive ion mode at atmospheric pressure. The detailed conditions have been described in the Supplementary Materials.

#### 4.4.8. Statistical Analysis

Statistical analyses were performed using standard nonparametric, correlation, and linear regression procedures. Continuous variables were summarized as median. Due to the substantial variability and non-normal distribution of the examined variables, between-group comparisons were performed using the Mann–Whitney U test.

The primary analysis compared tissue concentrations of E2, testosterone, and DHT between men with small prostates (TPV < 30 mL) and men with enlarged prostates (TPV ≥ 30 mL). A two-sided *p*-value < 0.05 was considered statistically significant. Data are presented as mean ± standard deviation (SD) or median with corresponding dispersion parameters, depending on data distribution and variance homogeneity.

Associations between TPV and tissue steroid concentrations were evaluated using correlation and linear regression analyses. Separate analyses were performed for the entire cohort and for the TPV-defined subgroups. Regression models were used to estimate the slope, intercept, correlation coefficient, F statistic, and corresponding *p*-value for the relationship between TPV and each measured tissue steroid. Correlation-matrix visualization was done by OriginPro 8 software, in order to explore the relationships among TPV, E2, testosterone, and DHT and to assess coordinated steroid accumulation within transition-zone prostate tissue.

Effect-size analyses were performed in R version 4.6.1 (R Foundation for Statistical Computing, Vienna, Austria) using the effect size (version 1.0.3), boot (version 1.3-32), and pwr (version 1.3-0) packages.

## 5. Conclusions

This study demonstrates that prostate enlargement is associated with coordinated intraprostatic accumulation of estradiol, testosterone, and dihydrotestosterone in transition-zone biopsy tissue. The simultaneous increase in all three steroids, together with their strong positive associations with TPV, supports the concept that benign prostatic enlargement is accompanied by a distinct local steroidal microenvironment rather than by isolated DHT excess alone.

The observed pattern is consistent with a model in which sustained local testosterone accumulation may provide the upstream substrate for parallel amplification of DHT-dependent androgenic signaling and E2-dependent estrogenic signaling. In this framework, progressive transition-zone enlargement may reflect a state of local prostatic hormonal hyperfunction, in which coordinated androgenic and estrogenic activity reinforces stromal–epithelial signaling, growth-factor production, tissue remodeling, and benign prostate growth.

Because steroid measurements were performed in transition-zone biopsy tissue and not in tumor-directed diagnostic cores, the observed hormonal profile should be interpreted primarily in relation to prostate enlargement rather than malignant tissue status. Although the cross-sectional design precludes causal inference, these findings provide a biologically coherent framework for future studies integrating tissue steroid quantification with histological compartment analysis, steroidogenic enzyme expression, AR and ER profiling, inflammatory and fibrotic markers, and longitudinal clinical outcomes. Such studies may determine whether coordinated accumulation of testosterone, DHT, and E2 defines a distinct hormonal phenotype of BPH with potential relevance for disease progression and treatment response.

## Figures and Tables

**Table 1:** Descriptive statistics of tissue estradiol (E2), testosterone, and dihydrotestosterone (DHT) in the studied groups.

TPV (mL)	*n*	Tissue E2			Tissue Testosterone			Tissue DHT		
		Mean ± SD (ng/g)	Range (ng/g)	Median (ng/g)	Mean ± SD (ng/g)	Range (ng/g)	Median (ng/g)	Mean ± SD (ng/g)	Range (ng/g)	Median (ng/g)
<30	35	0.014 ± 0.007	0.004–0.032	0.012	0.533 ± 0.154	0.197–0.961	0.517	3.663 ± 1.125	2.015–6.662	3.526
≥30	45	0.034 ± 0.018	0.012–0.086	0.031	0.906 ± 0.312	0.528–2.251	0.804	8.671 ± 3.525	3.828–24.645	7.903
Total	80	0.025 ± 0.017	0.004–0.086	0.020	0.743 ± 0.315	0.197–2.251	0.720	6.480 ± 3.704	2.015–24.645	5.742

TPV < 30, total prostate volume of small prostates; TPV ≥ 30 mL, large prostates; SD—standard deviation.

**Table 2:** Parametric and nonparametric effect-size estimates for between-group differences in hormone concentrations.

Parameter	Cliff’s Δ	95% BCa Bootstrap CI for Cliff’s Δ	Cohen’s d	95% CI for Cohen’s d
DHT	0.948	0.845–0.985	1.821	1.291–2.343
Testosterone	0.828	0.650–0.925	1.459	0.958–1.953
E2	0.803	0.625–0.906	1.407	0.910–1.897

DHT—dihydrotestosterone; E2—estradiol.

**Table 3:** Literature summary of estrogen hormones (total estrogens and estradiol) concentrations in normal prostate tissue, benign prostatic hyperplasia (BPH), and prostate cancer (PCa) tissue.

Reference	Total Estrogens (fmol/mg protein/h)				
	Normal Tissue	BPH	PCa	Method	Tissues Source
[70]	Stroma: 102 ± 17 PZ: 105 ± 26	Stroma: 223 ± 57 PZ: 175 ± 69		RIA	Cystoprostatectomy
	Estradiol (fmol/mg protein)				
[71]	Stroma: 2.2 ± 0.3 Epithelium: 4.4 ± 0.3	Stroma: 6.2 ± 0.8 Epithelium: 3.5 ± 0.4		RIA	Prostatectomy Prostate during donor nephrectomy
	Estradiol (pg/g)				
[68]	—	26.0 ± 3.5	—	RIA	TURP/prostatectomy
[73]	12.0	—	—	LC-MS/MS	TURP
[25]	47.3	—	—	RIA	RRP
[74]	22.6 *	15.5 **	—	GC-MS	Cystoprostatectomy/RRP
[75]	—	22.4	31.0	GC-MS	Cystoprostatectomy/RRP
Current research	14.0 ± 7.0	34.0 ± 18.0	—	LC-HRMS	TRUS biopsy

* Both zones, prostate volume < 50 g; ** Both zones, prostate volume ≥ 50 g; Abbreviations: BPH, benign prostatic hyperplasia; RIA, Radioimmunoassay; GC-MS, gas chromatography–mass spectrometry; LC-MS/MS, liquid chromatography–tandem mass spectrometry; LC-HRMS, liquid chromatography–high resolution mass spectrometry; PCa, prostate cancer; RRP, radical retropubic prostatectomy; TURP, transurethral resection of the prostate.

## Data Availability

All data supporting the findings of this study are available from the corresponding author upon reasonable request.

## Supplementary Materials

The following supporting information can be downloaded at: https://www.mdpi.com/article/10.3390/ijms27156785/s1.

## Abbreviations

The following abbreviations are used in this manuscript:

BHP	Benign Prostatic Hyperplasia
DHT	Dihydrotestosterone
E2	Estradiol
TZ	Transition Zone
TRUS	Transrectal Ultrasound
TPV	Total Prostate Volume
PCa	Prostate Cancer
PZ	Peripheral Zone
ERα	Estrogen Receptor Alpha
ERβ	Estrogen Receptor Beta
AR	Androgen Receptor
SHBG	Sex hormone-binding globulin
LC-HRMS	Liquid Chromatography High Resolution Mass Spectrometry
PSA	Prostate-Specific Antigen
QC	Quality Control
LOD	Limit of Detection
LOQ	Limit of Quantification
SD	Standard Deviation
%RSD	Relative Standard Deviation
MV	Mean Value
IS	Internal Standards
SPE	Solid Phase Extraction
DAD	Diode Array Detector
