# Supplementary material for: Elevated Estradiol and DHT Levels in the Prostatic Stroma as Key Drivers of Benign Prostatic Hyperplasia Pathogenesis
Source: Int J Mol Sci. 2026 Jul 29;27(15):6785. doi: 10.3390/ijms27156785 (PMC13466709; doi:10.3390/ijms27156785)
Supplement: Supplementary file 1 [file ijms-27-06785-s001.zip › ijms-4443250-supplementary.pdf]

Supplementary material

Table S1. Demographic and clinical characteristics of the patients included in the study.

Age	Weight (kg)	Height (cm)	AGA	TPV (mL)	TZPV (mL)	PSA (mg/ml)	BMI (kg/m²)	DHT (ng/g)	Testosterone (ng/g)	Estrogen (ng/g)	PH
**TPV < 30 mL, total prostate volume of small prostates**											
69	84	184	2	10	0.8	25.8	24.81	2.026	0.511	0.0132	m
66	80	180	5	12	3	24	24.69	2.122	0.961	0.0141	m
73	73	170	4	12	5	14.4	25.26	2.566	0.432	0.0074	m
86	60	165	4	13	1.7	207	22.04	3.658	0.611	0.0078	m
79	75	184	3	14	3.1	8.9	22.15	2.572	0.197	0.0282	b
83	60	165	1	14	0.75	6	22.04	2.015	0.343	0.0042	b
59	92	185	6	14	1.5	14.4	26.88	2.241	0.497	0.0086	m
66	74	170	3	14	5	38	25.61	4.125	0.733	0.0084	m
66	63	180	4	14	3	18.77	19.44	3.526	0.479	0.0152	m
66	63	180	4	14	3	18.77	19.44	3.3652	0.726	0.0094	m
67	62	165	3	15	3.6	8.98	22.77	2.348	0.356	0.0048	b
67	90	182	4	16	2.1	26.71	27.17	3.025	0.568	0.0117	m
65	86	172	6	17	2	5.35	29.07	2.812	0.429	0.0122	b
77	76	180	5	17	3.8	18.42	23.46	2.812	0.379	0.0085	m
72	95	180	2	17	1.8	17.11	29.32	3.856	0.552	0.0142	m
78	55	160	4	17	3.4	44.39	21.48	4.033	0.524	0.0168	m
74	68	170	3	18	3.8	7.07	23.53	4.214	0.524	0.0163	b
66	101	183	3	19	7.725	14.62	30.16	6.662	0.558	0.0233	m
63	83	180	2	19	4	30.8	25.62	5.063	0.418	0.0152	m
49	81	186	1	19	6	1500	23.41	3.276	0.402	0.0076	m
66	101	183	3	19	7.725	14.62	30.16	3.37	0.407	0.0097	m
74	91	182	4	19	3.7	16.89	27.47	3.277	0.402	0.0107	m
80	100	181	5	19	4	6.08	30.52	2.697	0.539	0.0115	b
83	67	180	6	21	4.8	11.57	20.68	3.741	0.425	0.0142	m
76	72	162	5	21	4.1	8.7	27.43	3.086	0.603	0.0132	b
69	90	170	5	22	3.5	75.1	31.14	3.881	0.815	0.0323	m
78	80	165	6	22	3.6	20.3	29.38	3.388	0.839	0.0313	m
64	102	182	5	22	2	6.63	30.79	3.785	0.632	0.0105	b
65	78	176	3	24	4.937	6.6	25.18	4.064	0.593	0.0092	b
71	93	173	1	25	5.5	13.8	31.07	4.669	0.471	0.0096	m
74	86	176	4	25	3.7	6.2	27.76	4.669	0.471	0.0096	b
59	88	186	2	25	10	32	25.44	4.669	0.471	0.0153	m
67	73	175	2	25	5	4000	23.84	5.632	0.717	0.0185	m
78	77	175	5	28	4.2	10.22	25.14	5.365	0.558	0.0101	m
73	61	160	1	29	4.9	19.68	23.83	5.598	0.517	0.0241	m
**TPV ≥ 30 mL, total prostate volume of large prostates**											

Age	Weight (kg)	Height (cm)	AGA	TPV (mL)	TZPV (mL)	PSA (mg/ml)	BMI (kg/m²)	DHT (ng/g)	Testosterone (ng/g)	Estrogen (ng/g)	PH
59	82	181	2	30	2.9	7.4	25.03	5.829	0.528	0.0263	b
85	95	175	3	30	5	8.65	31.02	5.896	0.986	0.0214	b
56	106	186	3	30	15.62	21.68	30.64	5.796	0.806	0.0123	m
64	74	175	2	30	10	46	24.16	5.265	0.803	0.0207	m
80	81	179	4	30	3.7	10.3	25.28	5.689	0.733	0.0211	m
68	87	174	2	30	10	10.3	28.74	5.896	0.764	0.0199	m
71	85	175	5	32	14.062	13.9	27.76	6.178	0.779	0.0178	m
70	90	175	3	32	8	11.61	29.39	4.985	0.689	0.0122	m
73	89	173	4	33	0.75	9.39	29.74	9.06	0.942	0.0532	b
67	110	180	6	33	4.4	9.26	33.95	6.526	0.563	0.0168	m
80	79	173	6	33	10	4.72	26.40	5.325	0.785	0.0217	b
68	87	183	3	35	19.389	10.6	25.98	6.214	0.912	0.0117	m
78	75	173	2	35	1	950	25.06	7.014	1.035	0.0325	m
77	82	176	5	36	10	8.83	26.47	7.222	0.597	0.0274	b
66	71	160	2	37	10.885	3.65	27.73	7.369	0.605	0.0164	b
77	80	172	5	37	11	15.54	27.04	3.828	0.609	0.0153	m
62	88	176	3	38	2.7	9.04	28.41	7.686	0.623	0.0315	b
68	100	190	3	38	5.5	6.58	27.70	5.077	0.806	0.0214	b
67	105	184	1	40	10	872.5	31.01	8.151	0.643	0.0274	m
70	71	167	3	40	10.177	2.52	25.46	7.736	0.956	0.0148	b
77	70	186	4	40	12	2000	20.23	8.214	0.952	0.0412	m
55	93	176	2	40	4.9	8.41	30.02	6.658	0.771	0.0256	b
82	65	168	3	41	16.26	8.07	23.03	8.423	0.657	0.0369	b
67	104	177	4	45	7.1	13.06	33.20	9.311	0.701	0.0365	m
74	58	176	6	47	0.9	1993	18.72	9.775	0.724	0.0156	m
75	75	176	4	48	8.4	10.8	24.21	10.007	0.735	0.0242	m
74	76	185	5	49	22.273	78.78	22.21	10.321	0.751	0.0347	m
85	60	170	2	50	0.75	1200	20.76	7.903	0.848	0.0474	m
73	90	176	5	50	16.8	9.65	29.05	10.471	0.758	0.0312	m
84	95	190	1	51	16.704	43	26.32	11.008	1.082	0.0386	m
77	70	178	2	52	30.725	6.29	22.09	10.872	0.778	0.0412	b
66	76	187	2	52	30.03	648.45	21.73	8.652	1.056	0.0432	m
65	91	176	2	53	34.632	8.63	29.38	7.663	0.803	0.0289	b
70	93	183	2	54	11	12.3	27.77	7.465	0.804	0.0313	m
65	78	179	1	56	10	7.68	24.34	6.332	0.892	0.0214	b
70	108	184	4	61	15	9.23	31.90	13.024	1.046	0.0321	b
81	82	165	4	69	18.5	39.18	30.12	11.681	0.976	0.0315	m
76	72	176	6	76	35.042	9.74	23.24	10.205	1.061	0.0415	b
71	87	184	4	79	14	8.7	25.70	12.402	1.091	0.0695	b
77	80	182	2	80	7.8	12.43	24.15	10.325	1.322	0.0514	m
76	77	174	4	94	11	9.55	25.43	12.684	1.263	0.0662	b
85	60	160	3	105	0.75	3033	23.44	8.325	1.562	0.0525	m
69	80	178	5	116	72.918	8.92	25.25	11.391	1.516	0.0741	b

77	80	180	5	180	0.75	6.83	24.69	24.645	2.251	0.0674	m
75	105	180	2	200	150	13.25	32.41	15.705	1.231	0.0861	m

TPV – total prostate volume, TZPV – transition zone prostate volume, PSA – prostate-specific antigen levels, BMI – body mass index, DHT – dihydrotestosterone, PH – prostatic hyperplasia; b – benign; m –malignant.

1. Materials and Methods

Limits of Detection and Quantification

Method linearity was established using methanolic mixtures of analytical standards over the selected concentration ranges, according to a previously published procedure [9]. Calibration curves were constructed by plotting analyte concentration against LC–HRMS response, using five concentration levels near the estimated LOD and five concentration levels covering the linear dynamic range. Regression analysis was per-formed using the least-squares method.

LOD and LOQ were calculated from the calibration curve constructed using concentrations around the expected detection limit, according to the following equations:

$$LOD = (3.3 \times SD)/a \quad (S1)$$

$$LOQ = (10 \times SD)/a \quad (S2)$$

where SD is the standard deviation of the response (expressed as the standard error of the intercept coefficient), and a is the slope of the calibration curve obtained by linear regression analysis using Microsoft Excel. The results of LOD and LOQ are given in Table S2.

Table S2. Regression equation, correlation coefficient (R^2) limit of detection (LOD) and quantification (LOQ) and recovery determined at three different concentrations.

Compound	Regression equation Y=A+BX									
	Slope (B)		Intercept (A)					Recovery		
	Value	Error	Value	error	R^2	LOD	LOQ	1	2	3
**Without matrix**										
**E2**	1.211×10^5	1.644×10^3	8.377×10^4	1.212×10^2	0.9981	0.036	0.119	105	103	97
**DHT**	3.093×10^5	4.138×10^3	3.842×10^3	12.626	0.9986	0.040	0.132	96	99	102
**Testosterone**	2.678×10^5	2.876×10^3	1.077×10^3	1.212×10^2	0.9974	0.039	0.132	104	99	107
**In matrix**										
**E2**	1.076×10^5	1.022×10^3	5.478×10^4	1.033×10^2	0.9956	0.044	0.145	93	99	96
**DHT**	1.276×10^5	2.018×10^3	3.443×10^3	22.626	0.9973	0.048	0.158	91	96	99
**Testosterone**	1.483×10^5	3.112×10^3	1.033×10^3	1.455×10^2	0.9954	0.051	0.165	90	94	101

E2-estradiol; DHT-dihydrotestosterone.

Recovery Study

Method recovery was evaluated at three concentration levels using TZ prostate tissue samples fortified with E2, DHT, and testosterone standards prior to tissue homogenization. The spiked samples were subsequently processed under the same experimental conditions as the study samples. The analytical recovery (%) for E2, DHT, and testosterone was calculated using the following equation:

$$\text{Recovery (\%)} = F/(F_0 + A) \times 100 \quad \text{(S3)}$$

where F is the measured concentration of the analyte in the spiked sample, F_0 is the concentration determined in the corresponding unspiked sample, and A is the added concentration of the analyte standard. The results of the recovery are given in TableS2.

Quality control of the calibrator was performed with ESI-L-Low Concentration Tuning Mix (G1969 85000), as described in our previously published study [9]. The mass ac-curacy of the 6210 TOF was 3 ppm, which provides a strong basis for confirming com-pound identities and for identifying and quantifying unknown compounds. The API Reference Mass Solution Kit (G-1969 85001) was used to determine the accuracy and precision of the instruments during analysis [9].

Accuracy and the precision of the method

Method accuracy and precision were evaluated using prostate tissue samples spiked with known concentrations of E2, DHT, and testosterone. A quality control mixture was added to each spiked sample, which was subsequently processed under the same experimental conditions as the non-spiked samples. The mean measured concentration and standard deviation were calculated for each analyte. Method precision was expressed as the relative standard deviation (%RSD), calculated using the following equation:

$$\%RSD = 100\% \times SD/MV \quad \text{(S4)}$$

where SD is the standard deviation and MV represents mean value of the measurement. The results obtained are presented in TableS3.

The accuracy was defined as the difference between the target and sampled values, that is:

$$\%\ \text{bias} = ((\text{measured value} - \text{nominal value})/\text{nominal value}) \times 100 \quad \text{(S5)}$$

Intra-day repeatability was evaluated using quality control mixture containing E2, DHT and testosterone. Five replicate injections were performed at 3 h intervals over a 1 day. Inter-day repeatability was assessed by analyzing five replicate injections of the same quality control mixture each day for a period of 2 weeks. The results of intra- and inter-day repeatability are presented in Table S3.

Table S3. Accuracy, precision, intra-, and inter-day repeatability of the method.

Compound	Mean value (ng/g)	SD (ng/g)	Precision (%RSD)	Accuracy (%bias)	Intra-day	Inter-day
					CV (%)	CV (%)
			E2			
**Level 1**	0.026	0.0008	3.077	2.443	3.986	4.076
**Level 2**	0.057	0.001	1.754	1.875		3.332
**Level 3**	0.122	0.004	3.279	3.087		2.987
			DHT			
**Level 1**	6.112	0.147	2.405	4.088	5.077	6.122
**Level 2**	12.987	0.412	3.172	4.324	5.122	5.653
**Level 3**	25.889	0.776	2.997	4.789	4.653	4.678
			Testosterone			
**Level 1**	0.312	0.007	2.244	3.118	4.077	3.976
**Level 2**	0.633	0.012	1.896	3.432	3.234	3.446
**Level 3**	1.432	0.032	2.235	3.774	3.665	3.111

E2 – estradiol; DHT–dihydrotestosterone; SD–standard deviation; RSD–relative standard deviation; %RSD– precision; CV–coefficient of variation.

Prostatic tissue samples obtained from ten patients with different total prostate volumes (TPVs) were homogenized and divided into two aliquots. One aliquot was spiked with internal standards (IS) before sample preparation, whereas the second aliquot was processed without the addition of internal standards.

The accuracy, precision, and recovery of the analytes were first assessed using standard solutions. To evaluate matrix effects, prostate tissue homogenates were used as the biological matrix, and the extracted samples were subsequently fortified with internal standards after solid-phase extraction (SPE). Matrix-matched calibration curves were constructed using the standard addition approach, and the corresponding validation parameters are summarized in Table S2.

Liquid chromatography-high resolution mass spectrometry (LC–HRMS)

The mobile phase comprised (A) 0.2% formic acid in ultrapure water and (B) acetonitrile. Elution was performed using the following linear gradient at a flow rate of 0.4 mL/min: 0–1.5 min, 5% B; 1.5–12 min, 5–95% B; 12–15 min, 95% B; 15–16 min, 95–5% B. Before analysis, the chromatographic system was equilibrated for 10 min under initial conditions (95% A and 5% B). Each sample (100 μL) was injected via the autosampler. Elution profiles were monitored with a DAD detector over the wavelength range 195–450 nm.

Ionization conditions were optimized using the following ESI parameters: capillary voltage 4.0 kV, gas temperature 350 °C, drying gas flow rate 12 L/min, nebulizer pressure 45 psig, and fragmentor voltage 70 V. The eluted analytes were nebulized and mixed with heated nitrogen prior to ionization. Mass spectra were recorded within an m/z range of 100–1500.
